# A brief review of the neurological manifestations of the coronavirus disease

**DOI:** 10.1186/s41983-020-00244-6

**Published:** 2020-11-23

**Authors:** Ajaya Kumar Ayyappan Unnithan

**Affiliations:** Muthoot Hospital, Kozhencherry, Pathanamthitta, Kerala 689641 India

**Keywords:** Coronavirus, COVID-19, Viral encephalitis, Encephalomyelitis, Stroke, Antiviral agents

## Abstract

**Introduction:**

It has been demonstrated experimentally that the coronavirus can enter the central nervous system through olfactory nerves and can even reach medulla. Neurological manifestations are observed more frequently in patients with coronavirus disease.

**Main text:**

The aim of the review is to seek evidence for infection of the nervous system by the human coronavirus and study the neurological manifestations of the coronavirus and its treatment. A search was done in PubMed, Google Scholar, CrossRef, and Scopus. There is evidence for the coronavirus infection of the nervous system from experimental studies, autopsy reports, and clinical studies. The virus can damage the nervous system either by direct viral damage to the neural cells or by immunopathology. Cerebral edema, neuronal degeneration, encephalitis, meningoencephalitis, acute disseminated encephalomyelitis, Guillain–Barré Syndrome, Bickerstaff’s brainstem encephalitis, Miller Fisher syndrome, polyneuritis, toxic encephalopathy, and stroke can occur. The coronavirus has been demonstrated in the cerebrospinal fluid by polymerase chain reaction technique in infected patients. The abnormalities of the coagulation system increase the risk of cerebrovascular disease. Chloroquine analogs, lopinavir/ritonavir combination, remdesivir, dexamethasone, and immunoglobulin have been shown to be useful for the treatment.

**Conclusion:**

There is substantial evidence for infection of the nervous system by the different strains of the human coronavirus. The coronavirus enters the nervous system either by the blood or from the olfactory nerves. The neurological diseases correlate with the severity of the coronavirus disease. The treatment is mainly supportive. The reports of patients with encephalitis, encephalomyelitis, and brainstem encephalitis show slow recovery. But a stroke has a high mortality.

## Introduction

The coronavirus outbreaks have caused the severe acute respiratory syndrome (SARS) in 2002–2003 and Middle East respiratory syndrome (MERS) in 2012 [1]. Now there is another outbreak of viral pneumonia from the city of Wuhan in China, which is spreading all over the world. The World Health Organization (WHO) named the new virus as the 2019 novel coronavirus (2019-nCoV) or severe acute respiratory syndrome coronavirus 2 (SARS-CoV-2) [2]. Now more than 42 million persons are affected worldwide and more than one million persons died. It can infect the respiratory, gastrointestinal, hepatic, and central nervous systems. Respiratory and gastrointestinal manifestations are common [3]. The initial symptoms are usually fever, malaise, and dry cough [1]. The Chinese Center for Disease Control and Prevention classified cases as mild (nonpneumonia and mild pneumonia), severe (dyspnea, tachypnea, blood oxygen saturation ≤ 93%, and/or lung infiltrates > 50% within 24 to 48 h), and critical ( respiratory failure, septic shock, and/or multiple organ dysfunction or failure) [4]. Acute diarrheal disease is another common manifestation [2, 3]. Liver enzymes are increasd [1]. Neurological symptoms also are observed in patients, in about more than one third [5, 6]. Headache, nausea, and vomiting are the mild neurological symptoms. Infection of the brainstem by the coronavirus has been demonstrated, and it is speculated as a mechanism partially responsible for the acute respiratory failure. It was shown in animal model that neurotropic coronavirus can enter the central nervous system (CNS) through the olfactory nerves [7].

## Main text

### Objective

The aim of the review is to seek evidence for infection of the nervous system by the human coronavirus and study the neurological manifestations of the coronavirus and its treatment. The aim includes the study of pathogenesis of the neurological diseases, investigations for diagnosis, treatment, and the prognosis.

## Methods

### Literature search

A search was done in PubMed, Google Scholar, CrossRef, and Scopus for articles on neurological manifestations of corionavirus. The keywords used were the Coronavirus, Coronavirus disease-19(COVID-19), Viral Encephalitis, Encephalomyelitis, Stroke, and Antiviral Agents.

### Eligibility criteria

All available articles on the subject were included. Articles containing experimental studies, protocols, clinical studies, case reports, series, comments, and reviews were studied.

### Data extraction

The articles were downloaded from the web. A qualitative analysis was done. The evidence was assessed from the experimental studies. The clinical data were tabulated. The pathogenetic mechanisms were sequenced. The treatment guidelines and outcomes were analyzed.

### Outcome measures

The outcomes were assessed in terms of severity of neurological disease, recovery time, mortality, and response to treatments such as pharmacotherapy, ventilation, and supportive measures.

## Results

A total of 51 articles are studied. Only those studies with promising positive results had been taken from the literature about treatment. The types of articles were one protocol, six reviews, four pathogenetic studies, seven experimental reports, sixteen clinical case and seires reports, ten pharmacological studies, and seven articles on immunotherapy. A descriptive analysis was done under the sections: pathogenesis, clinical profile, investigations, treatment, and prognosis. The points centered were evidence of infection of the nervous system by the coronavirus, routes of entry of the virus into the nervous system, neurological manifestations, laboratory and radiological evaluation, and evolving treatments.

## Discussion

### Pathogenesis

The viral strains studied in experimental studies are 229E and OC43 [8]. The demonstration of the coronavirus RNA in human brain autopsy samples is an evidence of its neuroinvasion. The virus may enter the CNS either by hematogenous dissemination or by neuronal retrograde dissemination [9]. It has been shown that the human coronavirus can disseminate from the olfactory bulb to the cortex and can also reach the medulla. The virus uses angiotensin-converting enzyme 2 receptors (ACE-2 receptors) in the epithelial cells of the respiratory and gastrointestinal tract for penetration [6]. The ACE2 receptors are present in the glial cells and neurons especially in the ventrolateral medulla and the nucleus of the tractus solitarius [6, 10]. ACE2 is found in the endothelial cells of the blood-brain barrier (BBB), and the virus can penetrate the BBB and invade the nervous system hematogenously [11]. The postmortem examination of a patient infected with severe acute respiratory syndrome coronavirus-2 (SARS-CoV-2) showed the virus in the neural and capillary endothelial cells in frontal lobe [12]. Experiments with cultures of human astrocytes, oligodendrocytes, and neuroglia pointed towards the persistence of infection of neural cell lines by human coronaviruses [13, 14]. It can damage the CNS either by direct viral damage to the neural cells or by immunopathology [9]. Acute disseminated encephalomyelitis (ADEM) due to the human coronavirus in a boy has been reported [15].

The human coronavirus infection of neuronal cell lines leads to an increased production of intracellular infectious viral particles, a stronger induction of the unfolded protein response (UPR), and an increased neuronal cell death by apoptosis [16]. Autopsy of SARS patients has shown edema and scattered red degeneration of the neurons in the brains [17]. The maladaptive inflammatory response causes excessive production of cytokines such as interleukin (IL)-1β, interferon (IFN)-γ, tumor necrosis factor (TNF)-α, IL-4, and IL-10 [11]. These disrupt the BBB and cause neuroinflammation. The cytokine storm syndrome due to the rise of proinflammatory cytokines beyond a threshold involves the CNS also along with the other systems and carries poor prognosis [18]. The abnormalities of coagulation system as evidenced by the D-dimer and platelet count increase the risk of cerebrovascular disease [19].

**Fig. 1:**
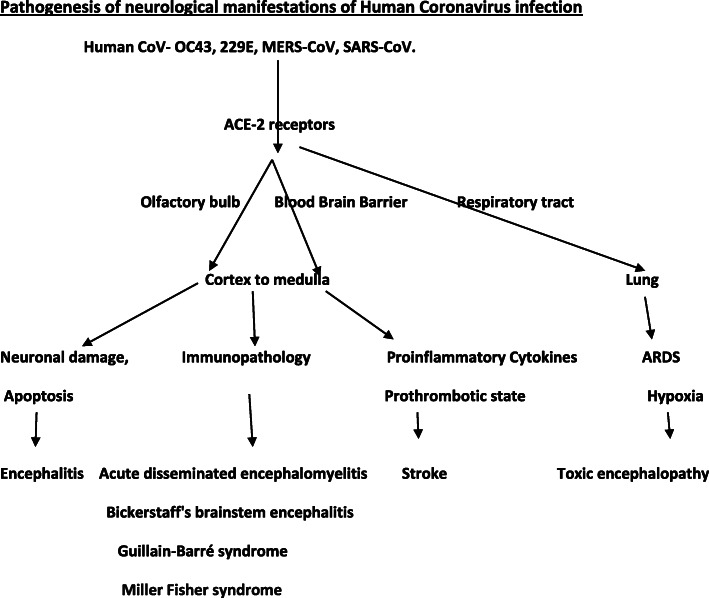
Pathogenesis of neurological manifestations of the human coronavirus infection. Human CoV human coronavirus, MERS-CoV Middle East respiratory syndrome coronavirus, SARS-CoV severe acute respiratory syndrome coronavirus, ACE-2 receptors angiotensin-converting enzyme-2 receptors, ARDS acute respiratory distress syndrome

The pathogenesis is depicted in Fig. 1.

### Clinical case profiles

The protocol of the World Health Organization ( WHO) mentions headache, seizures, alterarion of consciousness, and other neurological signs in the clinical profile of coronavirus infection [20]. Clinical features such dizziness, ataxia, impairment of taste, smell, and vision and neuralgia also are seen [19]. Olfactory and gustatory dysfunctions are observed in 30–80% in patients with mild COVID-19 [11].

Presentation like ADEM with numbness and weakness of the limbs, dysmetria was seen in a 15-year-old boy [15]. He had prior upper respiratory infection 1 week earlier. Polymerase chain reaction (PCR) test showed coronavirus OC43 in the cerebrospinal fluid (CSF) and nasopharyngeal secretions. A rise in antibody titer was also seen. Magnetic resonance imaging (MRI) showed multiple hyperintensities in the white matter of the brain and lesions in the cervical and thoracic segments of the spinal cord. Generalized tonic-clonic seizure in a lady with SARS has been reported [21]. She had fever, cough, and rigor. The X-ray of the chest showed patchy pneumonic consolidations and she was ventilated. She developed acute renal failure. She had seizure on the twenty-second day. CSF study was positive for coronavirus. Saad and colleagues reported a series of seventy patients infected by MERS coronavirus [22]. Nine patients had headache, eighteen had confusion, and six patients had seizures.

Inflammation of the brain by the virus leads to encephalitis with high fever, headache, vomiting, deterioration of consciousness, and seizures [23]. Moriguchi and colleagues described about a 24-year-old man who presented with headache, loss of consciousness, seizure, and neck rigidity [24]. The nasopharyngeal swab did not contain SARS-CoV-2 RNA. But it was detected in CSF. Magnetic resonance imaging (MRI) of brain showed hyperintensity along the wall of the inferior horn of the right lateral ventricle in diffusion-weighted images (DWI) and hyperintensity in the right mesial temporal lobe and hippocampus in fluid-attenuated inversion recovery (FLAIR), suggestive of meningo-encephalitis. Bernard and colleagues reported about two ladies who had meningo-encephalitis due to SARS-CoV-2 [25]. Both had mild respiratory and general symptoms. Nasopharyngeal swabs were positive. They developed severe neurological symptoms and one had a status epilepticus. The CSF examination results were compatible with viral meningo-encephalitis. Ye and colleagues described about a male patient with SARS-CoV-2 who had confusion due to encephalitis [26]. He had signs of meningeal irritation such as nuchal rigidity, Kernig’s sign, and Brudzinski’s sign and had extensor plantar response. Systemic toxemia and hypoxia can cause brain edema and toxic encephalopathy manifesting as delirium [22].

Signs such as ophthalmoplegia and limb weakness as in Bickerstaff’s brainstem encephalitis (BBE) or Guillain-Barré syndrome (GBS) also have been seen [27]. In a report by Kim and colleagues, four patients had neurological symptoms out of a cohort of twenty-three patients with MERS. All of the four patients had contact with MERS patients and had respiratory symptoms. The first patient, a 55-year-old male, had acute respiratory failure, septic shock, and multiorgan dysfunction syndrome (MODS). He was ventilated. He had bilateral ptosis and quadriparesis. The results of magnetic resonance imaging (MRI) and CSF studies were negative. He was presumed to have BBE. The second patient, a 43-year-old female, had respiratory and gastrointestinal symptoms and sepsis. She had paresis of both the lower limbs on the tenth day. The laboratory studies were negative and probably she had GBS. The other two patients had tingling of the hands and feet probably due to neuropathy.

Toscano and colleagues reported five cases of COVID-19-associated Guillain–Barré syndrome [28]. Four of them had positive nasopharyngeal swabs and one had positive serology test. The symptoms were lower-limb weakness and paresthesia in four patients and facial diplegia followed by ataxia and paresthesia in the other. Flaccid tetraparesis evolved within a period of 4 days in four patients. Three patients had to be ventilated. On electromyography, fibrillation potentials were present in four patients. MRI with gadolinium showed enhancement of the caudal nerve roots in two patients and enhancement of the facial nerve in one patient. Gutiérrez and colleagues described about two patients with confirmed SARS-CoV-2 in orophayryngeal swab, of whom one presented with Miller Fisher syndrome (MFS) and the other with polyneuritis cranialis [29]. A 50-year-old man had anosmia, ageusia, right internuclear ophthalmoparesis, right fascicular oculomotor palsy, ataxia, and areflexia. CSF study showed albuminocytologic dissociation. He had positivity for GD1b-IgG antibodies suggestive of MF syndrome. The second patient, a 39-year-old man, had ageusia, bilateral abducens palsy, areflexia, and albuminocytologic dissociation.

In the report by Mao and colleagues, out of two hundred fourteen patients hospitalized with COVID-19, seventy-eight (36.4%) had neurological manifestations [30]. Both ischemic and hemorrhagic stroke were reported. The patients with severe infection were more likely to develop neurologic manifestations such as acute cerebrovascular disease and impaired consciousness. The fact that some patients in this study had smell impairment supports the entry of the virus through the retrograde neuronal route.

In observational series study of fifty-eight patients admitted with acute respiratory distress syndrome (ARDS) due to COVID-19, forty-nine had neurological signs [31]. The study was done in two intensive care units (ICUs) in Strasbourg, France, by Helms and colleagues. Confusion was present in forty (69%) patients and agitation in twenty-six. Corticospinal tract signs (exaggerated tendon reflexes, ankle clonus, and bilateral extensor plantar reflexes) were present in thirty-nine patients (67%). MRI showed leptomeningeal contrast enhancement in eight patients, bilateral frontotemporal hypoperfusion in eleven patients, and ischemic stroke in three patients. Fifteen patients had dysexecutive syndrome (inattention, disorientation, or poorly organized movements in response to command) at the time of discharge.

The clinical manifestations and the neurological diseases reported are summarized in Tables 1 and 2.

**Table 1:** Clinical manifestations of coronavirus infection

Respiratory	Fever, dry cough, dyspnea, pneumonia, and severe acute respiratory syndrome
Gastrointestinal	Nausea, vomiting, and acute diarrheal disease
Hepatic	Elevation of liver enzymes
Neurological	Headache, vomiting, dizziness, seizures, ataxia, dysmetria, impairment of smell, impairment of taste, impairment of vision, neuralgia, numbness, tingling, weakness, ophthalmoplegia, facial diplegia, abducens palsy, areflexia, corticospinal tract signs, neck rigidity, and other signs of meningeal irritation, confusion, agitation, delirium, deterioration of consciousness, coma, and stroke
Systemic	Sepsis, shock, and multiorgan dysfunction syndrome

**Table 2:** Reported neurological diseases secondary to coronavirus

Reported neurological diseases	
Encephalitis Meningo-encephalitis Toxic encephalopathy Acute disseminated encephalomyelitis Bickerstaff’s brainstem encephalitis Guillain-Barré syndrome Miller Fisher syndrome Polyneuritis cranialis Neuropathy Ischemic stroke Hemorrhagic stroke	

### Investigations

One of the diagnostic tests is the detection of viral RNA in a clinical sample by reverse transcriptase-polymerase chain reaction (RT-PCR) [32]. The specimens usually tested are nasopharyngeal aspirate and stool. The other laboratory test is the serologic detection of an antibody to the coronavirus by immunofluorescent antibody (IFA) assay or by enzyme-linked immunosorbent assay (ELISA). The coronavirus has been demonstrated in the cerebrospinal fluid (CSF) by the PCR technology in infection of the CNS [15, 21]. An increase in cell counts and protein levels, especially immunoglobulins, is seen in CSF study of COVID-19 patients with neurological involvement [11].

Increased level of D-dimer in the serum indicates a high chance of ischemic stroke and computerized tomography (CT) or MRI scan should be done in case of symptoms [19, 30]. In the report by Mao and colleagues, the lymphocyte counts were lower for patients with CNS symptoms than without CNS symptoms, indicative of the immunosuppression [30].

MRI can show contrast enhancement of lesions in the meninges, brain, spinal cord, and nerve roots in patients with encephalitis, myelitis, meningitis, and Guillain–Barré Syndrome [11]. Perfusion abnormalities are prevalent in patients with COVID-19 with severe neurological symptoms.

### Treatment

Since there is no definitive treatment for coronavirus infection, management is mainly symptomatic and supportive care [1]. Oxygen therapy and mechanical ventilation are needed in case of severe respiratory infection [27]. Septic shock necessitates hemodynamic support. Treatment for encephalitis requires antiedema and anticonvulsant medications [19]. Intravenous immunoglobulin (IVIG) was given for GBS and MFS and has shown benefit [28, 29]. Anticoagulation is recommended for patients with ischemic stroke.

Although there is no evidence for pharmacotherapy for the coronavirus from any randomized controlled trial (RCT), around 300 clinical trials are ongoing [33]. Chloroquine analogs inhibit the entry of the virus into the cells and replication [34]. They are basic compounds and diffuse into the acidic cytoplasmic vesicles such as endosomes and lysosomes and increase the pH. The viruses require endosomal and lysosomal acidification and proteases for the replication. Chloroquine analogs also inhibit the production of several cytokines which contribute to the severity of viral infections. Chloroquine inhibited coronavirus replication in cell culture study [35]. In a clinical trial by Gautret and colleagues, hydroxychloroquine in a dose of 200 mg 8 hourly was found to reduce viral load in 20 patients with coronavirus disease-19 (COVID-19) [36]. Chloroquine can cause adverse effects such as cardiac arrhythmias and retinopathy [33].

In vitro studies demonstrated that the antiviral drugs such as lopinavir and ribavirin can inhibit the cytopathic effect of the coronavirus [37]. In a study of 41 patients by Chu and colleagues, administration of a combination of lopinavir (400 mg) and ritonavir (100 mg) orally 12 hourly resulted in milder course of the disease course and reduction in the viral load. The adverse effects of this combination are gastrointestinal distress and hepatotoxicity [33]. Ribavirin requires high dose to inhibit viral replication with high chance of hematologic and liver toxicity.

GS-5734, a nucleoside analog synthesized at Gilead Sciences, inhibited replication of coronavirus in human airway epithelial cell culture and reduced lung viral load in a mouse model [38]. It is a broad-spectrum antiviral agent, now known as remdesivir [39]. There is a report of successful treatment with remdesivir for pneumonia due to novel coronavirus [40].

The effect of steroids in COVID is controversial. The literature does not support the routine use of corticosteroids in COVID-19 [41]. So C and colleagues noted a benefitial effect of methylprednisolone in patients with acute respiratory distress syndrome (ARDS) caused by COVID-19 [42]. The randomized evaluation of COVID-19 therapy (RECOVERY trial) has recently shown that dexamethasone reduced a 28-day mortality among those receiving invasive mechanical ventilation or oxygen [43].

Currently, there is no effective vaccine against the virus. But the spike (S) glycoprotein is used in most of the experiments as the major inducer of neutralizing antibodies, to develop a vaccine [44]. RNA vaccines are also in trial [45]. Live attenuated virus vaccine and DNA vaccine are also being developed [46]. Covalescent plasma (CP), taken from recovered patient, with good immunoglobulin antibody titer, is a form of passive immunotherapy [47, 48]. Initial evidence favors its use. Vero cell studies have shown the efficacy of various interferons (IFN) such as alpha, beta, and gamma subtypes in inhibiting the replication of coronavirus [49, 50].

### Prognosis

The main factors predisposing to severe illness are old age and associated comorbidities [51]. The reports of patients with encephalitis, encephalomyelitis, Bickerstaff’s brainstem encephalitis, Guillain-Barré syndrome, Miller Fisher syndrome, neuropathy, and polyneuritis show slow recovery over months [15, 21, 27]. But coma and stroke are associated with high mortality [30]. In the report by Mao and colleagues, it was difficult to assess the outcome of those with neurologic manifestations, since most of the patients were hospitalized at the time of analysis.

### Limitation of the study

The pandemic is still continuing and evolving. So there are no conclusive analytic studies. The ongoing clinical studies are expected to give good results. Future research can be planned based on the current knowledge.

## Conclusion

There is substantial evidence for infection of the nervous system by the different strains of the human coronavirus. There is evidence from experimental studies, autopsy report, clinical studies, CSF studies, and MRI findings. Neurological manifestations occur in more than one third of patients with COVID. The coronavirus may enter the nervous system either by hematogenous dissemination or from the olfactory nerves to the cortex. It can cause neurological disease due to direct viral infection or by immunopathology. Cerebral edema, neuronal degeneration, encephalitis, meningo-encephalitis, acute disseminated encephalomyelitis, Guillain–Barré Syndrome, Bickerstaff’s brainstem encephalitis, Miller Fisher syndrome, polyneuritis, toxic encephalopathy, and stroke can occur. The neurological diseases correlate with the severity of COVID. Treatment is mainly supportive. Chloroquin analogs, lopinavir/ritonavir combination, remdesivir, and immunoglobulin have been shown to be useful for the treatment. There is a recent report of efficacy of dexamethasone. Slow recovery is seen in a neurological coronavirus disease. But stroke carries a high mortality.

## Data Availability

PubMed, Scopus, and Google Scholar.

## Abbreviations

SARS: Severe acute respiratory syndrome
MERS: Middle East respiratory syndrome
WHO: World Health Organization
2019-nCoV: 2019 novel coronavirus
SARS-CoV-2: Severe acute respiratory syndrome coronavirus 2
CNS: Central nervous system
COVID-19: Coronavirus disease-19
ACE-2 receptors: Angiotensin-converting enzyme 2 receptors
BBB: Blood-brain barrier
ADEM: Acute disseminated encephalomyelitis
UPR: Unfolded protein response
IL: Interleukin
IFN: Interferon
TNF: Tumor necrosis factor
PCR: Polymerase chain reaction
CSF: Cerebrospinal fluid
MRI: Magnetic resonance imaging
DWI: Diffusion-weighted images
FLAIR: Fluid-attenuated inversion recovery
BBE: Bickerstaff’s brainstem encephalitis
GBS: Guillain-Barré syndrome
MFS: Miller Fisher syndrome
MODS: Multiorgan dysfunction syndrome
RT-PCR: Reverse transcriptase-polymerase chain reaction
IFA: Immunofluorescent antibody assay
ELISA: Enzyme-linked immunosorbent assay
CT: Computerized tomography
IVIG: Intravenous immunoglobulin
RCT: Randomized controlled trial
ARDS: Acute respiratory distress syndrome
CP: Convalescent plasma
